# Complete corneal ring (MyoRing) implantation versus MyoRing implantation combined with corneal collagen crosslinking for keratoconus: 3-year follow-up

**DOI:** 10.1007/s10792-017-0593-4

**Published:** 2017-06-15

**Authors:** Guzel Bikbova, Gyulli Kazakbaeva, Mukharram Bikbov, Emin Usubov

**Affiliations:** 1 Ufa Eye Research Institute, Ufa, Russia; 2 Department of Ophthalmology and Visual Science, Chiba University Graduate School of Medicine, Inohana 1-8-1, Chuo-ku, Chiba, Chiba 260-8670 Japan

**Keywords:** Cornea, Keratoconus, Corneal collagen crosslinking, MyoRing

## Abstract

**Purpose:**

To estimate the effectiveness of complete corneal ring (MyoRing) implantation compared with MyoRing implantation combined with corneal collagen crosslinking (CXL) for keratoconus treatment for 36 months follow-up.

**Design:**

Retrospective cohort study.

**Materials and methods:**

MyoRing implantation was performed in a series of 78 patients (80 eyes) with keratoconus II–III Amsler classification, of these 39 eyes had MyoRing implantation combined with CXL. Implantation of a MyoRing in the corneal pocket was performed using a PocketMaker microkeratome and corneal intrastromal implantation system. During CXL, riboflavin solution 0.1% was injected into the corneal pocket through the incision tunnel and standard surface UVA irradiation (370 nm, 3 mW/cm^2^) was then applied from 5-cm distance for 30 min.

**Results:**

Significant improvements in uncorrected distance visual acuity and corrected distance visual acuity were observed for both groups, which was relatively better 12 months after procedure in MyoRing alone group; however, in 36 months there was no difference between groups. Keratometry was reduced in both groups; after MyoRing implantation for 8.45 D and MyoRing + CXL for 9.43 D, the spherical equivalent decreased from 8.45 to 7.72 D and from 9.43 to 6.25 D, respectively. The cylinder decreased to 3.33 D with MyoRing alone and to 3.31 D with MyoRing + CXL. Corneal thickness remained nearly unchanged (from 433.69 ± 38.76 to 434.21 ± 34.98) in MyoRing group and decreased from baseline (from 426.93 ± 46.58 to 401.24 ± 39.12 µm) in MyoRing + CXL group 36 months postoperatively, which corresponds with pachymetry reduction after conventional CXL.

**Conclusion:**

Both MyoRing implantation and MyoRing combined with CXL were effective for treating keratoconus. At 36 months, there were slightly better outcomes in MyoRing + CXL group; however, in MyoRing alone group visual and refractive outcomes were stable overtime.

## Introduction

Keratoconus is a noninflammatory corneal disease characterized by progressive thinning of the cornea that is accompanied by ectasia [1]. Changing the volume of to the peripheral cornea by ring segment implantation is reported to be useful for improving visual acuity and reducing the corneal steepening associated with keratoconus [2].

Different types of corneal segments may be used for keratoconus treatment, such as Intacs (Addition Technology, Inc.), Ferrara ring (Ferrara Ophthalmics Ltd.), and Keraring (Mediphacos Ltd.). A complete intrastromal ring, called MyoRing (Dioptex, GmbH, Linz, Austria), suggested by Albert Daxer in 2007, is relatively new and had been demonstrated to treat keratoconus safely and effectively [3–5]. With a surgical system known as the corneal intrastromal implantation system (CISIS), the MyoRing (flexible full-ring implant) is inserted into the corneal pocket, using a high-precision microkeratome (PocketMaker microkeratome) [3–5].

Corneal collagen crosslinking (CXL), introduced by Wollensak et al. [6], has become a standard treatment for progressive keratoconus [6–9] to slow or possibly stop the progression of a disease. The standard technique involves epithelial removal to enable appropriate penetration of riboflavin into the stromal tissue where highly reactive oxygen species trigger formation of crosslinks that consist of intrafibrillary and interfibrillary covalent bonds [6].

The formation of the stromal pocket during MyoRing implantation offers the opportunity for simultaneous introduction of 0.1% riboflavin into the pocket followed by UVA irradiation to provide combined treatment for patients with progressive keratoconus [5]. The results of the combination of CXL with MyoRing implantation with 12 months of follow-up were reported by Studeny et al. [7].

Nobari et al. [8] presented the comparative study of MyoRing alone and MyoRing implantation for patients who previously had CXL within 12 months before MyoRing implantation.

Some reports demonstrated that ring segments have the disadvantage of resulting in loss of visual acuity in the long term [9, 10]. In contrast, it was reported that MyoRing implantation itself may stop the progression of disease due to the continuous ring shape design and its ability to strengthen the biomechanical property of cornea, and its ability to stabilize the corneal thickness [2, 11], e.g., Daxer in his study found that for ring segments and incomplete rings, the strengthening factor was 1.0 and that a intracorneal continuous complete ring (MyoRing) had a strengthening factor of up to = 3.2, because it is a continuous full-ring implant, with no disruption of continuity along its circumference, suggesting that it may act as an artificial limbus and provide biomechanical support to the cornea [12, 13].

In a recent report by Daxer, it was indicated that no significant progression was observed after MyoRing treatment during an average follow-up period of 5 years [13].

The aim of this study was to estimate the effectiveness of complete corneal ring (MyoRing) implantation alone compared with MyoRing implantation combined with corneal collagen crosslinking (CXL) for keratoconus treatment with 36 months of follow-up.

## Materials and methods

### Study group and protocol

This retrospective cohort study was performed in Ufa Eye Research Institute from January 2010 to March 2015 and included 3 years of follow-up.

All patients provided informed written consent. The study was approved by the ethics committee of Ufa Eye Research Institute (reference number 462.29.9369) following the tenets of the Declaration of Helsinki and local laws regarding research involving human subjects.

Inclusion criteria were age older than 18 years, a diagnosis of keratoconus, intolerance of contact lenses or glasses, and documented progression of a disease. This progression was defined by the following changes over the course of 1 year: an increase of the steepest *K* by 1.0 diopter (D) or more in the manifest cylinder, or an increase of 0.5 D or more in the manifest spherical equivalent (SE) refraction by repeated keratotopography ODP-scan ARK-1000 (Nidek, Japan).

Exclusion criteria were minimal pachymetry of less than 380 μm, a history of previous ocular pathology or ocular surgery, pregnancy or breastfeeding, and corneal scarring.

Of 115 patients who had MyoRing implantation alone or MyoRing implantation combined with CXL, 35 of them were not able to attend follow-up examinations and therefore they were excluded from the study. This study included 80 eyes from 78 patients with progressive keratoconus of grade II–III according to the Amsler classification (without stromal scarring).

### Measurements and devices

Patients were examined at baseline and at 12, 24 and 36 months post-MyoRing implantation + CXL. At each follow-up visit, a standard examination was done to assess uncorrected distance visual acuity (UDVA), corrected distance visual acuity (CDVA), refractometry, keratometry, corneal topography (ODP-scan ARK-1000 Nidek, Japan), and pachymetry (Visante OCT, Carl Zeiss, Germany). To create a stromal pocket for further MyoRing implantation, the PocketMaker microkeratome PocketMaker (Dioptex GmbH, Linz, Austria) was used.

During CXL, pachymetry measurements were taken with a handheld ultrasound pachymeter (SP-3000, Tomey, Japan).

To control the safety of the procedure, endothelial cell density was counted in all patients, and corneas were scanned using laser scanning confocal microscope. Images of the endothelium and cornea were acquired with a confocal scanning laser ophthalmoscope (Heidelberg Retina Tomograph III/Rostock Corneal Module; Heidelberg Engineering GmbH, Germany). Endothelial cell density was assessed using the software provided by the system.

The CXL device was used at a distance of 5 cm with irradiation of 3 mW/cm^2^ (UFalink, Russian Federation). Before each treatment, a calibration was performed to confirm the correct UVA emission level.

### Surgical technique

Implantation of a MyoRing in the corneal pocket was performed by using a PocketMaker microkeratome, as described elsewhere [3–5]. The device uses a guided, vibrating diamond blade to create a stromal pocket 9 mm in diameter at a 300-μm depth via a 4–5-mm wide corneal tunnel. In the group of MyoRing combined with CXL group, 0.1% sterile riboflavin solution was then continuously injected for 3 min into the corneal pocket through the incision tunnel via standard 0.3-mm cannula.

The efficiency of riboflavin penetration into the corneal stroma was checked by slit-lamp examination on a dark blue cobalt filter. An intense yellow glow in the anterior and posterior stroma confirmed riboflavin distribution throughout the cornea.

Standard surface UVA irradiation (370 nm, 3 mW/cm^2^; UFalink, Russian Federation) was then applied at a 5-cm distance for 30 min. During the UVA exposure, injection of the riboflavin solution into the pocket occurred every 10 min. After UVA irradiation, a flexible MyoRing intracorneal implant was inserted into the corneal pocket as described elsewhere [3–5]. The diameters of the rings used in this study were 5 mm with a thickness of 240, 280, or 320 μm, according to the nomogram recommended by the manufacturer. Central corneal thickness recordings were performed throughout the UVA irradiation and shown were 400 μm or higher. The MyoRing placement was adjusted in three patients 2 days after surgery because of insufficient refractive improvement after the initial implantation.

### Statistical analysis

Decimal visual acuity was converted to the logarithm of the minimal angle of resolution (logMAR).

Statistical analysis was performed using GraphPad Prism 4 software for Macintosh (version 4.0c, GraphPad Software, Inc.). Data were recorded as mean ± standard deviation (SD). Baseline measurements (preoperative and 1 year postoperative) between groups were compared using a two-tailed paired Visual acuity and refractive test. Statistical significance for differences between preoperative and postoperative data was defined as *P* < 0.05 for all cases.

## Results

The patients included 54 men (70%) and 24 were women (30%), aged 18–48 years (average 27.06 ± 2.02). Table 1 shows the baseline characteristics.

**Table 1:** MyoRing implantation for keratoconus, baseline characteristics (*n* = 78)

Parameter	MyoRing alone (*n* = 41)	MyoRing + CXL (*n* = 39)
UDVA, LogMAR	0.90 ± 0.28	1.06 ± 0.24
CDVA, LogMAR^†^	0.45 ± 0.28	0.49 ± 0.26
Pachymetry thinnest point (μm)	433.69 ± 38.76	426.93 ± 46.58
SE (D)	−9.03 ± 4.07	−7.86 ± 4.52
Cylinder (D)	−4.87 ± 3.13	−3.98 ± 2.85
K1 (D)	48.67 ± 3.44	48.73 ± 3.87
K2 (D)	54.21 ± 6.27	53.83 ± .5.54
K Av (D)	51.56 ± 5.42	50.25 ± 4.32

*UDVA* uncorrected distance visual acuity; *CDVA* corrected distance visual acuity; *SE* spherical equivalent; *K*1 corneal dioptric power in the flattest meridian; *K*2 corneal dioptric power in the steepest meridian; *K* *Av* mean corneal power; *CCT* corneal thickness at thinnest location; *D* diopter; *CXL* corneal crosslinking

^†^Spectacle corrected

Values are mean ± SD

Group 1 with MyoRing implantation alone included 41 eyes, group 2 included 39 eyes that had MyoRing combined with CXL.

No intraoperative complications were observed. In the MyoRing alone group, five patients (12.1%) reported glare and night-vision problems postoperatively. Additional 1% pilocarpine eye drops were prescribed for 1 month postoperatively. In the MyoRing + CXL group, nine patients (23.1%) developed slight stromal edema at 1 month after surgery, which was resolved within 3 months postoperatively.

**Fig. 1:**
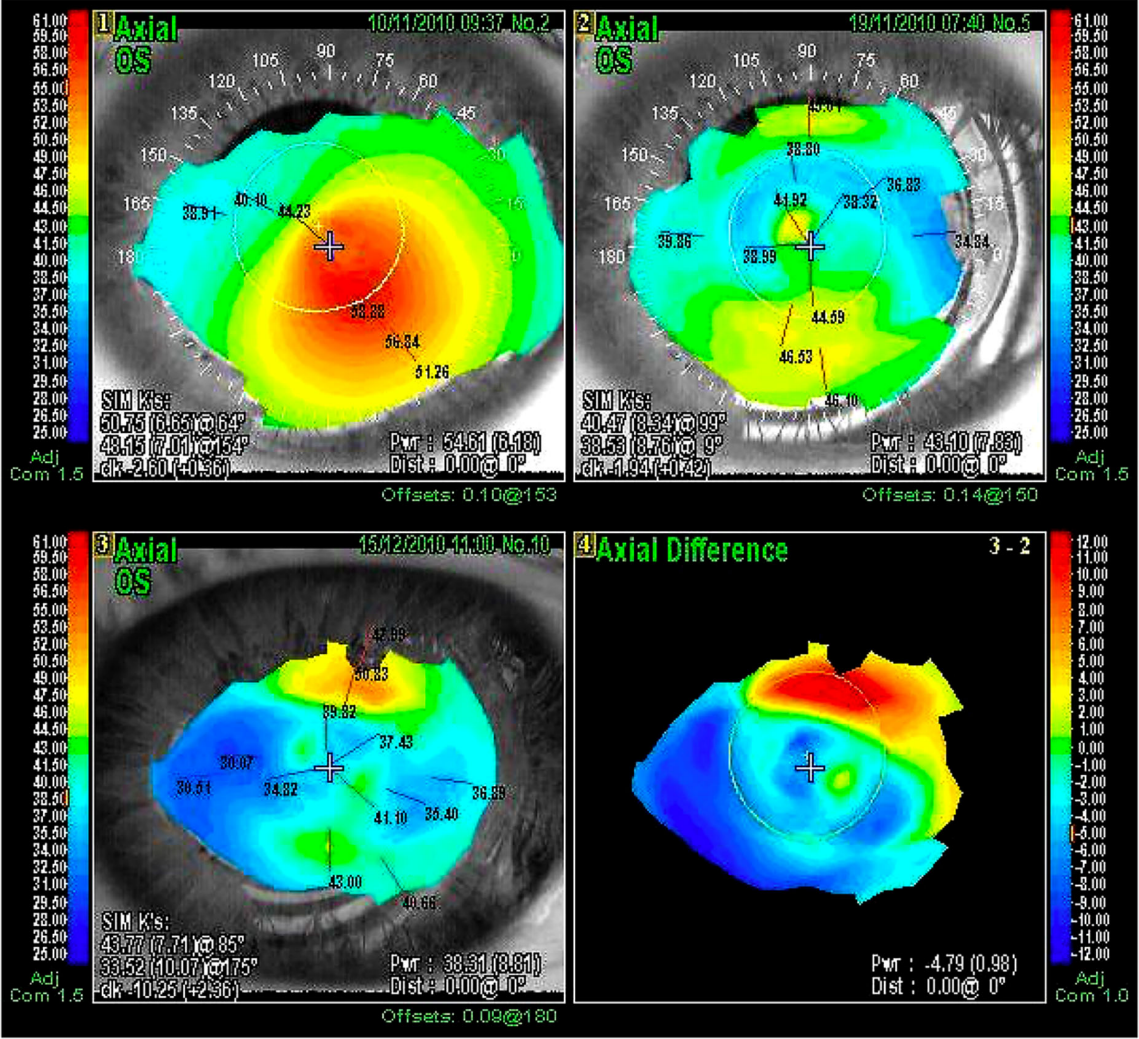
*1* Preoperative topography. The central cornea is irregular, with the steepest point in the temporal inferior quadrant. *2* Postoperative topography. The central cornea with a bow-tie pattern and fairly regular astigmatism compared to image 1. *3* Postoperative topography after MyoRing placement was adjusted 0.5 mm. This resulted in an additional decrease of corneal refraction and astigmatism. *4* Differences in corneal refraction between postoperative MyoRing placement and adjustment (−4.79 D)

Three patients underwent adjustment of the MyoRing placement 2 days after surgery because of insufficient refractive improvement after the initial implantation. Reposition of the MyoRing within 0.5 mm allowed an additional decrease of keratometry up to 4 D (Fig. 1). Table 2 summarizes the visual and refractive outcomes over time. All parameters improved significantly as a result of implantation in both groups.

**Table 2:** Postoperative data, 36 months after MyoRing + CXL. Visual acuity and refractive outcomes are shown

Parameter	36 months post-op	*P* interaction
UDVA, LogMAR		
MyoRing	0.31 ± 0.28, *P* < 0.0001	0.2463
MyoRing + CXL	0.30 ± 0.23, *P* < 0.0001	
CDVA, LogMAR^†^		
MyoRing	0.28 ± 0.26, *P* = 0.0491	0.5136
MyoRing + CXL	0.23 ± 0.23, *P* < 0.0001	
Spherical equivalent (D)		
MyoRing	−1.31 ± 3.15, *P* < 0.0001	0.2818
MyoRing + CXL	−1.61 ± 3.18, *P* = 0.0279	
Pachymetry thinnest point (μm)		
MyoRing	434.21 ± 34.98, *P* = 0.0931	0.1391
MyoRing + CXL	401.24 ± 39.12, *P* = 0.0395	
Corneal astigmatism (D)		
MyoRing	−1.54 ± 2.25, *P* = 0.0414	0.5112
MyoRing + CXL	−0.67 ± 1.89, *P* = 0.0441	
K1 (D)		
MyoRing	41.64 ± 4.73, *P* = 0.0151	0.0491
MyoRing + CXL	40.12 ± 4.11, *P* = 0.0179	
K2 (D)		
MyoRing	45.01 ± 2.65, *P* = 0.00506	0.0913
MyoRing + CXL	42.79 ± 3.15, *P* = 0.01179	
K Av (D)		
MyoRing	43.11 ± 2.68, *P* < 0.000001	0.0488
MyoRing + CXL	40.82 ± 3.11, *P* = 0.01	

*UDVA* uncorrected distance visual acuity; *CDVA* corrected distance visual acuity; *K*1 corneal dioptric power in the flattest meridian; *K*2 corneal dioptric power in the steepest meridian; *K* *Av* mean corneal power; *CCT* corneal thickness at thinnest location; *D* diopter; *CXL* corneal crosslinking

Values are mean ± SD

^†^Spectacle corrected

**Fig. 2:**
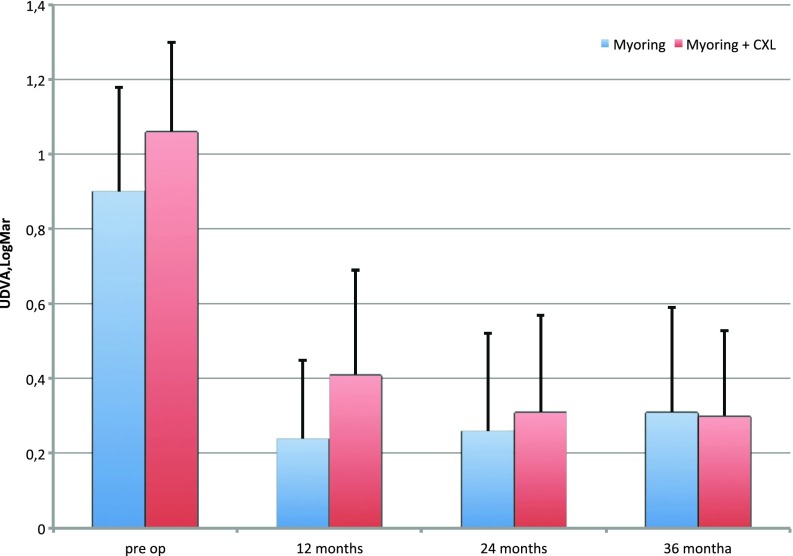
Uncorrected distance visual acuity (UDVA) over time [logarithm of the minimal angle of resolution (logMAR)]. The *error bars* represent standard deviation (SD)

Significant improvements in UDVA and CDVA were observed in both groups, but relatively better improvement was seen 12 months after procedure in MyoRing alone. However, in 36 months there was no difference between groups (Fig. 2).

**Fig. 3:**
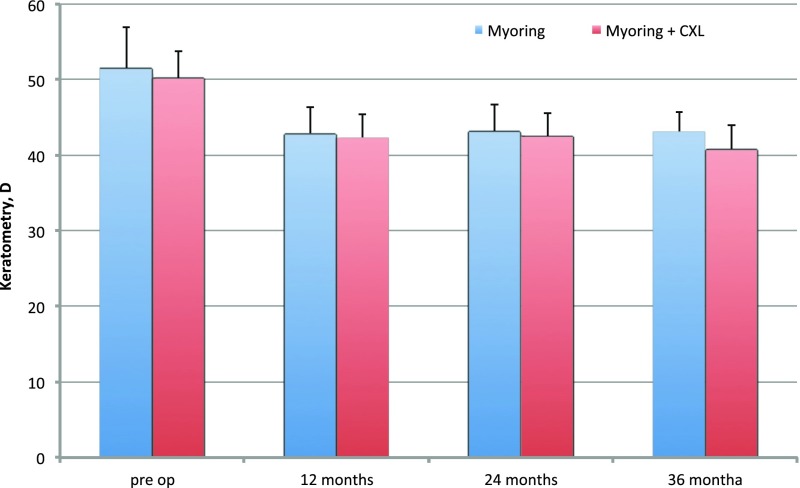
Keratometry reading over time. The *error bars* represent standard deviation (SD)

Significant central corneal flattening was observed in both groups. Mean keratometry decreased to 8.45 D after MyoRing alone implantation and to 9.43 D after MyoRing + CXL (Fig. 3). Statistically significant reductions of spherical equivalent (SE) and corneal astigmatism (CA) were observed overtime, SE decreased to 7.72 D with MyoRing alone compared to 6.25 D with MyoRing + CXL, and CA was 3.33 D with MyoRing alone and 3.31 D with MyoRing + CXL. Further improvements in keratometry, SE and CA had been observed in MyoRing + CXL group; however, statistical significance was not reached (between 12 and 24 months *P* = 0.0511, and between 12 and 36 months *P* = 0.6083 for keratometry, between 12 and 24 months *P* = 0.4036, and between 12 and 36 months *P* = 0.594 for SE, between 12 and 24 months *P* = 0.2986, and between 12 and 36 months *P* = 0.7015 for CA). There was no statistical significant difference between two groups over the time for all variables except for keratometry (Table 2).

Corneal thickness at the thinnest point remained nearly unchanged (433.69 ± 38.76–434.21 ± 34.98) in MyoRing group and decreased from baseline values (426.93 ± 46.58–401.24 ± 39.12 µm) in the MyoRing + CXL group 36 months postoperatively, which corresponds with pachymetry reduction after conventional CXL [14, 15].

It was not possible to assess the demarcation line because the stromal pocket was formed at a depth of 300 µm. However, confocal microscopy clearly showed that the stroma had a “honeycomb” appearance, with a reduced number of keratocyte nuclei. The maximum depth of penetration measured from the surface of the epithelium was 237–302 µm. At about 6 months postoperatively, the corneal stroma had regained its normal configuration [16–19].

Confocal microscopy also demonstrated the hyperreflectivity of the epithelial layer in the MyoRing + CXL group, and some slight hyperreflectivity of epithelial cells in the MyoRing alone group. Disruption and irregularity of subepithelial nerves were observed in both groups 3 months after surgery, and the recovery was achieved within 6–12 months. Additionally, in the group of MyoRing alone group the hyperreflective keratocytes in the anterior and mid stroma were noticed at 3 months postoperatively. By 6 months, the stroma had regained its normal configuration. Reflective amorphous structures, located to inner and outer edges of the ring, were observed in some patients in both groups at 36 months after surgery. The haze in the MyoRing + CXL group was observed as a hyperdensity of extracellular tissue, which had been resolved within 6–12 months. The endothelial cell density remained nearly unchanged in both groups (2846 ± 67 cells/mm^2^ and 2912 ± 73 cells/mm^2^, respectively).

## Discussion

Implantation of the MyoRing permits individualized treatment of keratoconus through control of the ring position as well as its diameter and thickness. The pocket technique allows postoperative adjustment of the MyoRing postoperatively to achieve the best position for it [3]. The corneal pocket is created at a depth of 300 μm, and it has a diameter of 9 mm. Compared to intracorneal ring segment nomograms, the MyoRing nomogram is simple and does not require consideration of the location and cone type. Our study showed that MyoRing implantation significantly improved both UDVA and CDVA and significantly reduced SE and keratometry values.

No intraoperative complications were observed during this study. Postoperatively 12.1% of patients had night-vision problems and halo postoperatively; however, these issues were corrected with pilocarpine 1% eye drops for 1 month.

Repositioning of the MyoRing was performed in two cases. Adjustment within 0.5 mm can result in much better outcomes, in contrast to ring segment surgery, which involves the rings pacing in circular tunnels; thus, repositioning of ring segment can be achieved by changing the course of the tunnel [20].

The possibility of adjusting the position, thickness and diameter of the ring and the reversibility of surgery provide the surgeon with advantages for achieving optimal results for the patient.

However, there are some reports for some disadvantages of long-term ring segments implantation [9, 10]. Alio et al. [9] indicated that statistically significant regression up to 3.36 D in keratometric readings occurred in progressive cases from 6 months up to 5 years.

Daxer calculated the strengthening factor for the characterization of different ring-shaped corneal implant designs and concluded that the small lamellar incision during the pocket creation technique does not affect the biomechanical stability of the corneal tissue [12]. In addition, the geometry of the complete ring with full mechanical strength of the material along the entire circumference may be biomechanically considered as a further (artificial) limbus, in contrast to ring segments, because their incomplete ring geometry has no strengthening effect on the cornea [12].

Our study showed stable outcomes in the group for the MyoRing implantation alone within 36 months. Collagen crosslinking allows stabilization of disease. Results over the past 10 years are available confirming the effectiveness of CXL for halting the progression of keratoconus; however, additional visual rehabilitation is needed, such as contact lenses, intracorneal ring segments, or phakic intraocular lenses [21, 22]. Additionally, different transepithelial approaches exist [23–26] to avoid epithelial debridement during CXL and to reduce pain and discomfort during the early postoperative period; therefore, the formation of the stromal pocket during MyoRing implantation is a significant advantage for simultaneous introduction of 0.1% riboflavin into the stromal pocket for further UVA irradiation. Daxer et al. [5] described the combined technique of MyoRing implantation and CXL with the intrastromal application of riboflavin into the pocket at once. Several studies have confirmed that after MyoRing implantation, the parameters remained unchanged within 12 months [13, 27]. Our study demonstrated slight improvement 24 and 36 months after the combined procedure corresponding to the results of conventional CXL. Studeny et al. used combination of MyoRing with CXL and a 12-month follow-up for a group of 22 eyes with keratoconus [8] and noted improvements in the results between 1 month and 1 year after surgery similarly to our study. Slight improvements after CXL are well-known findings after 1–2 years; therefore, it can be concluded that further improvements after 24 months after the combined procedure are due to the effects of CXL [8], as confirmed by our study. Nobari et compared MyoRing alone and MyoRing implantation for patients who underwent CXL within 12 months before MyoRing implantation [7]. The follow-up period was 12 months. They did not find significant differences between groups except for CDVA, and better outcomes were found for the MyoRing alone group. In our study, CXL procedure was performed during MyoRing implantation, and similar to the Nobari et al. study, UDVA and CDVA were slightly better for those in the MyoRing alone group. However, at 36 months there were no differences between groups. The difference in the CDVA can be explained by the haze formation in the group who had combined procedure [26]. However, in our study, slight differences in keratometry and pachymetry values were found, corresponding to conventional CXL results, but no statistical significant differences were found between groups 36 months after surgery except for keratometry (*P* = 0.0488).

Reports of the combined technique using ring segments for CXL are available. Coskunseven et al. reported the results of combined KeraRing implantation and CXL. Their group 1 underwent ICRS first and then CXL, and their group 2 underwent CXL first and then ICRS. The interval between treatments was approximately 7 months. They found that these methods combined provided better outcomes, especially when CXL was performed after ICRS implantation [28]. Renesto et al. [29] reported a prospective study with a 2-year follow-up period; they found no difference between patients with ICRS implantation alone and patients treated with CXL followed by ICRS implantation 3 months later. However, Chan et al. [21] performed a retrospective comparative study of Intacts alone compared to Intacs implantation followed by CXL and demonstrated that combined surgery resulted in significantly greater reductions in the cylinder (2.73 vs. 1.48 D) and the maximum *k* value (1.94 vs. 0.89 D) than did Intacs implantation only.

There are some advantages using CXL with MyoRing implantation in contrast with ring segments. Using CXL with the pocket creation technique, which uses a corneal pocket for intracorneal riboflavin application, keeps the epithelium intact, avoids the postoperative discomfort that typically follows conventional CXL, and allows fast and complete saturation of the cornea with riboflavin solution. The 9-mm corneal pocket corresponds to the diameter of the treated cornea used in the standard CXL procedure.

Our study showed that MyoRing implantation alone and MyoRing combined with CXL can be an efficient approach for treating keratoconus. Both methods were safe and effective for treating moderate and severe keratoconus. After 36 months, there had slightly better outcomes in MyoRing + CXL group; however, visual and refractive outcomes for the MyoRing alone group were stable over time. However, a longer follow-up and randomized prospective studies with bigger number of patients are needed to confirm the effectiveness of both methods.

Limitation of the current study was that there was no evaluation of aberrations, the small group sizes, non uniformity of patients at each stage of keratoconus.
